# Online validation of DC/TMD Axis II questionnaires for assessing temporomandibular disorders

**DOI:** 10.22514/jofph.2026.040

**Published:** 2026-05-12

**Authors:** Hande Uzunçıbuk, Maria Maddalena Marrapodi, Aida Meto, Marco Di Blasio, Vincenzo Ronsivalle, Diana Russo, Marco Cicciù, Giuseppe Minervini

**Affiliations:** ^1^Department of Orthodontics, Faculty of Dentisty, Trakya University, 22030 Edirne, Türkiye; ^2^Department of Woman, Child and General and Specialized Surgery, University of Campania "Luigi Vanvitelli", 80138 Naples, Italy; ^3^Saveetha Dental College and Hospitals, Saveetha Institute of Medical and Technical Sciences (SIMATS), Saveetha University, 600077 Chennai, India; ^4^Department of Dentistry, Faculty of Dental Sciences, University of Aldent, 1007 Tirana, Albania; ^5^Department of Biomedical, Surgical and Dental Sciences, University of Milan, 20122 Milan, Italy; ^6^Department of Biomedical and Surgical and Biomedical Sciences, University of Catania, 95123 Catania, Italy; ^7^Multidisciplinary Department of Medical-Surgical and Dental Specialties, University of Campania "Luigi Vanvitelli", 80138 Naples, Italy

**Keywords:** DC/TMD, Orthodontics, Reproducibility of results, Surveys and questionnaires, Temporomandibular joint disorders

## Abstract

**Background**: The purpose of this study was to evaluate the consistency and dependability of the online and traditional analog versions of the Diagnostic Criteria for Temporomandibular Disorders (DC/TMD) Axis II Evaluation Questionnaires. **Methods**: In a randomized manner, 100 participants completed the questionnaires in both traditional analog and online formats using Google Forms. Internal consistency was tested using the Cronbach’s alpha coefficient, with the criterion being >0.700. Intraclass correlation coefficient (ICC) with 95% confidence interval (CI) was applied for the calculation of the analog–online test agreement levels. The mean differences between the online and analog questionnaires were used to calculate effect sizes (ES). **Results**: Cronbach’s alpha coefficients of all scales exceeded 0.700, suggesting satisfactory internal consistency. The ICC values ranged between 0.950 (95% CI: 0.926–0.966) and 0.999 (95% CI: 0.999–0.999), suggesting strong agreement between formats. All the effect sizes fell within the small to medium range, congruently supporting consistency between methods. **Conclusions**: The present findings indicate that the online version of the DC/TMD Axis II Evaluation Questionnaires can be a reliable instrument for evaluating symptoms in respect to temporomandibular disorders.

## 1. Introduction

Epidemiologic studies have shown that the most common oral disorders among all age groups in every population are the TMDs. According to a systematic review, the prevalence of TMDs varies from 3% to 80%, contingent on the population under investigation and the diagnostic standards used [1]. Studies indicate that 60% to 75% of individuals experience at least one symptom of TMDs in their lifetime, while approximately 35% exhibit clinical signs of the disorder [2]. Because the way TMDs are shown in different groups is so different, standardized assessment tools are definitely needed. The DC/TMD (Diagnostic Criteria for Temporomandibular Disorders) Axis II Questionnaires can be a valid tool in the standardized assessment of the prevalence of TMD. Orthodontics also proves essential in treating TMDs through the correction of discrepancies of the jaw and the occlusion, relieving pressure on the temporomandibular joint, hence the alleviation of symptoms related to the disorder. Studies show that orthodontic treatment can lower the possibility of developing TMDs. This makes standardized instruments like the DC/TMD Axis II Questionnaires even more important for accurate assessment and monitoring.

Prevalence of TMDs also exists in the pediatric population, yet there has not been much documented regarding this age group in comparison with adults. For example, a Saudi Arabian children survey study demonstrated a prevalence of TMDs at 4.5%, evidencing that TMDs are not exclusive to adults [3]. A study from Brazil found that most research is done on adults, and there aren’t as many studies that use standardized diagnostic criteria on younger people [4]. It is essential to extend the research focus to children and adolescents in order to better understand the development of TMDs in early life and what might be playing a part.

Hormonal, genetic, psychosocial, and environmental factors are all part of the complicated etiology of TMD. The hormonal causes, in addition, explain the higher prevalence of TMDs in females, the group that also has higher prescription rates of TMD-related pain treatment [5]. Stress, anxiety, as psychosocial factors, are attributed to the development of TMDs as well as the aggravation of symptoms [1]. Owing to the complicated nature, the DC/TMD Axis II Questionnaires derives significance in the evaluation of the biological as well as the psychosocial aspect of TMDs, thus supplementing the entire diagnosis as well as treatment approach.

Two axes make up the DC/TMD, of which Axis I is the determination of physical diagnoses and Axis II in the assessment of psychosocial status and disability associated with pain. Axis II of the DC/TMD incorporates self-report questionnaires to assess the psychosocial and psychological consequences associated with TMDs-related pain [6, 7, 8, 9, 10]. Its inclusion of the Axis II instruments of the DC/TMD system is crucial, as patients’ psychological can be determined by using the equation and behavioral health, in an effort to determine factors that contribute to distress and functional impairment. The DC/TMD Axis II evaluation Questionnaires comprise several tools, namely the Graded Chronic Pain Scale (GCPS), the Jaw Functional Limitation Scale (JFLS), the Patient Health Questionnaire (PHQ), the General Anxiety Disorder (GAD) and the Oral Behaviors Checklist [10, 11]. The utilization of the DC/TMD Axis II Evaluation Questionnaires holds significant value in evaluating the psychosocial implications of TMDs and formulating treatment strategies [11, 12, 13]. The correlation between Axis I with Axis II is fundamental in characterizing TMDs patients, as a comprehensive understanding of both physical and psychosocial factors is essential for effective management. Despite this importance, the literature highlights the need for further exploring the interplay between these two axes. Digital tools can support research and clinical practice, all the more so in consideration of the necessity of using a biopsychosocial model of TMDs.

Management of DC/TMD Axis II Questionnaires assessment can be accomplished either in analog or in online techniques [14]. To confirm the accuracy and reliability of these diagnosis tools are therefore of the highest importance. Validation studies are required for determining whether the instruments provide reliable and uniform measurements, since this has significance in their applications in clinical work [15, 16, 17]. By determining the reliability and validity of the tele-medium, validation studies enhance confidence in tele-assessments, making them ready for applications in treatment planning, as well as in the daily care of patients [18, 19]. These studies thus significantly enhance the quality of scientific work and the expansion of practical applications in clinical work [20, 21, 22, 23, 24].

Validating the online version of the DC/TMD Axis II is essential to ensure its reliability and accuracy, as telehealth and remote assessments gain prominence in improving accessibility for patients unable to attend in-person evaluations. Given the increasing reliance on telehealth, validating an online version of the DC/TMD Axis II is essential to make TMDs assessments more accessible and convenient for patients who may face barriers to in-person visits. This approach could improve compliance and data collection, allowing for broader and more frequent patient evaluations without compromising diagnostic quality. This study focuses on Axis II due to its use of self-reported psychosocial measures, which are more adaptable to online assessment than Axis I’s clinical examinations. The online format has been preferred to the traditional formats to facilitate patient compliance, increase data collection efficiency, and decrease demands on resources. Nevertheless, such constraints as patients’ familiarity with digital technology and data security signify the need for strenuous verification in an aim to achieve diagnostic quality. This study serves as an important step toward integrating validated digital tools in clinical practice, especially as remote healthcare options expand.

The Turkish versions of the DC/TMD Axis II assessment forms were compared in this study between online and analog (paper-and-pencil version) formats. This study set out to compare the online and traditional analog versions of the DC/TMD Axis II Evaluation Questionnaires in order to assess their consistency and dependability. According to the null hypothesis, the online DC/TMD Axis II assessment for TMD diagnosis is just as accurate and dependable as the traditional analog version.

## 2. Materials and methods

### 2.1 Participants and procedure

The study’s sample recruitment took place in February 2023, as indicated by the approval date of the ethical clearance (Protocol Number: 18, Date: 11 January 2023). The time frame for sample recruitment in the study was structured around a one-week interval between the administration of online and analog questionnaires. That time interval was chosen to eliminate recall bias without jeopardizing reliability of response.

The justification for the use of 100 participants in the present study originates in the COSMIN (COnsensus-based Standards for the selection of health Measurement INstruments) guidelines, as at least 100 participants are recommended for validation studies. This decision was further supported by previous studies that successfully used comparable sample sizes for online questionnaire validations [25]. Participants were selected randomly from the general public with no prior screening or diagnosis for TMDs. To increase the validity of the results, a randomization process was used to reduce selection bias and make the study population more representative of the general population. Individuals who had difficulty reading or writing were excluded from the study.

Either an online or conventional analog questionnaire was filled out by the participants. Group assignment was done randomly using a computer-generated number sequence (via https://www.random.org) to ensure fair and unbiased distribution between the two formats. A group of 50 participants initially completed the questions and answers in a traditional analog format, while another group of 50 participants completed the questionnaires in an online format through the use of the Google Form service. After completing the online questionnaires, participants were asked to complete the analog version, and those who finished the analog versions had to finish the online ones.

Appropriate time intervals should minimize recall bias and increase the reliability of repeated measurements in accordance with the COSMIN guidelines. The time interval between the administration of the online and analog questionnaires was one week in accordance with similar studies in the literature [26, 27]. One-week interval study design aims at reducing potential biases or other external variables that would affect the subjective outcomes within this time frame. The reason for selecting a one-week interval between evaluations, instead of conducting both on the same day with a one-hour break, was to ensure an adequate inter-session clearance interval. To prevent participants’ responses from being biased by their memory of the initial questions, the study aimed to evaluate the questionnaire formats’ reliability over a short period without the interference of instant recall. Hence, the present study was carried out with a group of 100 participants as in other previous studies [25, 28].

In total, the study included 100 individuals, with 100 completed analog DC/TMD-Axis II evaluation questionnaires and 100 completed online questionnaires, constituting the dataset used for analysis.

### 2.2 Measures

#### 2.2.1 Sociodemographic information

Participants were asked to answer sociodemographic questions such as age, gender, education, occupation, and marital status.

#### 2.2.2 DC/TMD-Axis II evaluation questionnaires

##### 2.2.2.1 The graded chronic pain scale (GCPS)

Graded Chronic Pain Scale (GCPS) has emerged as one of the standard instruments for ascertaining the severity of chronic pain and the level of disability in a person’s daily living. Based on the degree of disability and the intensity of the pain, it divides chronic pain into five grades. Grade 0 has no pain, while Grade I has minimal intensity of pain with minor disability. Grade II has a high intensity of pain but minimal disability. While Grade IV is characterized by very severe pain and profound disability, Grade III also involves moderate to high levels of pain but still results in significant functional limitations [10, 29].

##### 2.2.2.2 Patient health questionnaire-4 (PHQ-4)

The Patient Health Questionnaire-4 (PHQ-4) has 4 items that are self-administered to screen depression and anxiety symptoms. Two are depression items, and the other two are the anxiety items. Participants were asked to rate how often they experienced each symptom over the past two weeks, using a 4-point scale ranging from 0 (never) to 3 (almost every day). Overall range (0–12) indicates increased psychological distress as the number increases. When the depression subscale or the anxiety subscale reaches 3 or higher, clinical evaluation may be warranted for depression or generalized anxiety disorder [10, 30].

##### 2.2.2.3 Patient health questionnaire-9 (PHQ-9)

PHQ-9 has become widely utilized depression severity scale and screening tool. It contains nine items, of which each one corresponds to a central symptom of depression. Respondents are instructed to indicate how often they had experienced each of the symptoms in the previous two weeks, aided by the same 4-point scale of the PHQ-4. The total score can be between 0–27, and larger values indicate greater depression It is interpreted as follows: 0–4 represent minimal or no depression, 5–9 represent mild depression, 10–14 are linked to moderate depression, 15–19 represent moderately severe depression, and 20–27 represent severe depression [10, 31].

##### 2.2.2.4 Patient health questionnaire-15 (PHQ-15)

It screens for the existence and the severity of 15 common physical (somatic) symptoms, including pains, gastrointestinal problems, cardiopulmonary symptoms, and neurological symptoms. Participants are asked how much they are bothered by each of the symptoms in the previous two weeks with a 3-point scale: 0 if “not bothered at all”, 1 if “bothered a little”, and 2 if “bothered a lot”. The overall burden of the somatic symptoms is the scale total, with a range of 0 to 30. A total score between 0 and 4 suggests little to no symptoms, 5 to 9 points to a mild level, 10 to 14 indicates a moderate level, and scores between 15 and 30 reflect a high level of symptom severity. Larger values can be associated with the diagnosis of the somatic symptom disorder or other connected medical disorders [10, 32, 33].

##### 2.2.2.5 General anxiety disorder-7 (GAD-7)

The seven questions in the GAD-7 assess the frequency of generalized anxiety symptoms, and individuals are asked to rate the frequency of their experiences on a scale from 0 to 3 (0 = not at all, 1 = several days, 2 = more than half the days, and 3 = nearly every day). Scores are interpreted as follows: 0–4 suggests little to no anxiety, 5–9 indicates mild anxiety, 10–14 points to a moderate level, and 15–21 signals severe anxiety [10].

##### 2.2.2.6 Jaw functional limitation scale-8 (JFLS-8)

The Jaw Functional Limitation Scale-8 (JFLS-8) is a short, self-reported questionnaire that helps assess how temporomandibular disorders (TMDs) and similar conditions impact jaw movement and a person’s overall quality of life. It includes 8 items that explore limitations in everyday activities such as chewing, speaking, and facial expressions [10, 32, 34].

##### 2.2.2.7 Jaw functional limitation scale-20 (JFLS-20)

The JFLS-20 consists of 20 questions that individuals answer to evaluate the limitations and difficulties they experience related to jaw function and pain. These are questions about multiple functions of the jaw as well as how TMDs affect daily living. The JFLS-20 contains items about pain, function of the jaw, eating, speaking, and general quality of life associated with the function of the jaw. Respondents rate the frequency and severity of their experiences on a scale, which may vary depending on the specific version of the JFLS-20 in use [10, 32, 34].

##### 2.2.2.8 Oral behavior checklist (OBC)

The Oral Behavior Checklist (OBC) is an assessment tool to evaluate parafunctional habits and behaviors in individuals. These behaviors can include clenching or grinding of teeth, tongue-thrusting, mouth breathing, nail-biting and other habits that may have a relationship to TMDs. The OBC questionnaire includes 21 questions. Each response to OBC is assessed on a 5-point Likert-type scale. The score varies from 0 to 84, with classifications of none, low and high. Only a high score represents the risk factor for TMDs [10].

### 2.3 Data integrity and quality control measures for analog-to-digital conversion

A number of quality control procedures were put in place to guarantee data integrity and reduce errors when converting analog questionnaire responses to digital format. Two independent researchers (HU, AM) manually entered the responses into a digital spreadsheet, enabling the identification and resolution of discrepancies between entries through a reconciliation process. Next, another researcher (MMM) also verified the digital data against the original analog questionnaires to verify accuracy. Any inconsistencies identified during this verification process were logged, reviewed and corrected to align the digital data with the original responses. A predefined standardized coding system was used to keep data points consistent and lower the chance of mistakes. We also used automated scripts to find errors in numeric data fields and mark unlikely values, like outliers or missing data, for further review. All researchers responsible for data entry and verification received comprehensive training and calibration to ensure a consistent understanding of the procedures and to minimize the likelihood of human error. Additionally, the manually digitized data were compared with the digitally recorded responses from the online format using intraclass correlation coefficients (ICCs) and effect size analysis to validate consistency and reliability. These processes aided in making the results of the study valid and accurate, particularly in the comparison of the answer between the analog and the online.

### 2.4 Statistical analysis

The statistical analyses in this study were conducted using the NCSS (Number Cruncher Statistical System) 2007 Statistical Software package program, developed in Kaysville, UT, USA. In addition to utilizing descriptive statistical approaches such as calculating the mean, standard deviation, frequency and percentage distributions, the Cronbach’s alpha coefficient of the scales was assessed. Agreement between the analog version of the scales and the online versions was established through the intraclass correlation coefficient (ICC), at a 95% confidence interval (CI). To evaluate the mean values between the two formats, a paired *t*-test was employed. Additionally, effect sizes (ES) were calculated based on the mean differences between the results of the analog version and the online version of the questionnaires. Internal reliability of the scales was approximated through Cronbach’s alpha, a scale of 0 to 1 that indicates the extent to which items in a scale are connected. Values of 0.7 or above are well accepted for the purpose of good reliability. Cohen’s effect size (ES) was used in determining the magnitude of the difference between the two formats. It is calculated by taking the variation of between-group means divided by the standard deviation. An effect size of 0.2, 0.5, and 0.8 are, respectively, deemed to be small, medium, and large by Cohen’s criterion. Cronbach’s alpha in general makes the measurement of the scale’s reliability straightforward, while Cohen’s ES reflects the strength or the significance of the group difference [35]. Correlation reveals the direction of the relationship between variables, as well as the strength, while the effect size takes into consideration how meaningful, or how important that relationship is. Even if the outcome of the test of a relationship between variables has statistical significance, an outcome of a small effect might indicate that the practical difference between the groups remains minimal. In this context, our study anticipated small to medium ES values, reflecting minor differences between the two formats. Our study compared the responses to the online and analog formats of the DC/TMD-Axis II assessment questionnaires, which are universally validated and translated into multiple languages. We specifically compared different formats of the same questionnaires. Therefore, based on norms of Cohen’s criterion of ES, we anticipated that the ES would be between 0.20–0.50. This range reflects the expectation of only minor differences, suggesting that participants’ responses remain consistent between the analog and online formats of the same questionnaire.

The study design and analysis were guided by the COSMIN framework, which provides rigorous criteria for evaluating the reliability and validity of health-related measurement tools; including internal consistency (Cronbach’s alpha ≥0.7), test-retest reliability (intraclass correlation coefficient with 95% confidence intervals), and measurement equivalence (effect size analysis), ensuring methodological rigor and adherence to these standards [36, 37].

## 3. Results

Of the 100 participants, 73% were female, with an average age of 25.15 ± 9.41 years, and 27% were male, with a slightly wider age range (±11.85 years). 64% had only finished high school, whereas 36% had a university degree. 40% of participants were employed, while 60% were unemployed. In terms of marital status, 64% were unmarried and 36% were married.

Cronbach’s alpha values for all administered scales exceeded 0.700, indicating high internal consistency. This confirms the reliability of the instruments used to measure the intended psychological and functional constructs. Notably, the PHQ-15 scale showed the lowest values (0.790 for online and 0.754 for analog formats), indicating moderate reliability for assessing somatic symptom severity. In contrast, scales like the JFLS-20 (with Cronbach’s alpha values above 0.940) and the GAD-7 (exceeding 0.890) demonstrated strong internal consistency, ensuring reliable performance across both online and analog formats (Table 1). These findings support the overall reliability of the questionnaires used. Lower Cronbach’s alpha values derived for the PHQ-15 may be the result of some diversity in the way the participants responded—due to the complexity or subjective nature of some items.

**Table 1.** The assessment of the DC/TMD-Axis II evaluation questionnaires’ Cronbach’s alpha coefficient (to assess measurement reliability).

Cronbach’s Alpha Coefficient		Online	Analog
GCPS			
	Characteristic Pain Intensity	0.838	0.821
	Interference	0.853	0.844
	Total Score	0.867	0.861
JFLS-8		0.871	0.852
JFLS-20			
	Global	0.874	0.868
	Mastication	0.895	0.873
	Mobility	0.912	0.924
	Communication	0.955	0.977
	Total Score	0.952	0.947
PHQ-4		0.887	0.847
PHQ-9		0.867	0.858
PHQ-15		0.790	0.754
GAD-7		0.909	0.892
OBC		0.886	0.867

*GCPS: Graded Chronic Pain Scale; JFLS: Jaw Functional Limitation Scale; PHQ: Patient Health Questionnaire; GAD: General Anxiety Disorder; OBC: Oral Behavior Checklist.*

The degree of consistency and repeatability of the data between the online and paper-and-pencil versions was determined using the Intraclass Correlation Coefficient (ICC). ICC of the total scores of all the questionnaires ranged between 0.950 (95% CI: 0.926–0.966) to 0.999 (95% CI: 0.999–0.999), all significantly greater than the acceptable limit of 0.700. These results confirm excellent agreement between the two versions, proving that the participants responded in a constant fashion despite the fact that they responded either in the analog version or in the online version of the questionnaire [38] (Table 2).

**Table 2.** The assessment of the Analog and online DC/TMD-Axis II evaluation questionnaires’ ICC and 95% CI values.

Scale	Subscale	ICC	95% CI
GCPS			
	Characteristic Pain Intensity	0.999	(0.999–0.999)
	Interference	0.999	(0.998–0.999)
	Total Score	0.950	(0.926–0.966)
PHQ-4		0.994	(0.991–0.996)
PHQ-9		0.996	(0.994–0.997)
PHQ-15		0.970	(0.956–0.980)
GAD-7		0.994	(0.991–0.996)
JFLS-8		0.990	(0.985–0.993)
JFLS-20			
	Global	0.997	(0.995–0.998)
	Mastication	0.995	(0.992–0.996)
	Mobility	0.997	(0.996–0.998)
	Communication	0.995	(0.992–0.996)
	Total Score	0.998	(0.997–0.999)
OBC		0.995	(0.992–0.996)

*ICC: Intraclass Correlation Coefficient; CI: Confidence Intervals; GCPS: Graded Chronic Pain Scale; PHQ: Patient Health Questionnaire; GAD: General Anxiety Disorder; JFLS: Jaw Functional Limitation Scale; OBC: Oral Behavior Checklist.*

The exceptionally high ICC values observed in this study can be attributed to several factors. First, the participants’ familiarity with the questionnaire content during repeated administrations likely contributed to the consistency of responses. Second, the inherent robustness and standardization of the validated scales ensured reliable measurement across both formats. These findings suggest that the scale design decreases variation with respect to format.

Strong agreement between the analog and the online formats, shown by the high intraclass correlation coefficient (ICC) values, highlight the consistency of participants’ responses between the two versions, thus validating the online format as an acceptable alternative for taking tests at a distance. The result has special significance in clinical and research applications, where convenience, as well as accessibility, are important—serving to ensure diagnostic accuracy, no matter what format the questionnaire has been given. A comparison of the mean scores between online and analog responses revealed statistically insignificant differences for most items and scales. However, exceptions were observed in individual questions across some scales:

On the GCPS scale, a significant difference was identified in question 7, with the analog mean being higher than the online mean (*p* < 0.05). The effect size for this question was small (0.020), aligning with the acceptable range for small to medium levels (Table 3).

**Table 3.** The assessment of the participants’ replies obtained from both the online and analog versions of the GCPS questionnaire with their effect sizes.

GCPS	Online	Analog	Difference	*p*	ES
Question 1	2.92 ± 5.71	2.91 ± 5.71	0.01 ± 0.10	0.320	0.005
Question 2	0.72 ± 1.65	0.73 ± 1.68	−0.01 ± 0.10	0.320	0.005
Question 3	1.59 ± 2.59	1.59 ± 2.59	0.00 ± 0.00	1.000	0.000
Question 4	1.06 ± 1.81	1.06 ± 1.81	0.00 ± 0.00	1.000	0.000
Question 5	0.72 ± 3.15	0.72 ± 3.15	0.00 ± 0.00	1.000	0.000
Question 6	0.82 ± 1.80	0.83 ± 1.82	−0.01 ± 0.10	0.320	0.005
Question 7	0.87 ± 1.91	0.91 ± 1.90	−0.04 ± 0.20	0.045*	0.020
Question 8	0.79 ± 1.77	0.83 ± 1.70	−0.04 ± 0.49	0.417	0.003
Characteristic Pain Intensity	11.23 ± 17.33	11.27 ± 17.39	−0.03 ± 0.33	0.320	0.005
Interference	8.00 ± 18.23	8.23 ± 17.91	−0.23 ± 1.33	0.095	0.044
Total Score	3.29 ± 5.86	3.52 ± 7.16	−0.23 ± 2.85	0.422	0.035

GCPS: Graded Chronic pain Scale; ES: Effect Size. Paired t-test; *p < 0.05.

Question 2 of the PHQ-4 scale was significantly different, while questions 1, 5, 7, 9 and 10 of the PHQ-15 scale showed significant differences. Despite these differences, all effect sizes remained within the acceptable range (Table 4).

**Table 4.** The assessment of the participants’ replies obtained from both the online and analog versions of the PHQ-4, PHQ-9 and PHQ-15 questionnaires with their effect sizes.

Scale		Online	Analog	Difference	*p*	ES
PHQ-4						
	Question 1	1.10 ± 0.82	1.08 ± 0.86	0.05 ± 0.22	0.055	0.026
	Question 2	0.89 ± 0.91	0.98 ± 0.89	−0.09 ± 0.29	0.002*	0.047
	Question 3	0.96 ± 0.76	0.99 ± 0.77	−0.03 ± 0.22	0.181	0.009
	Question 4	0.98 ± 0.83	0.98 ± 0.84	0.00 ± 0.14	0.998	0.000
	PHQ-4 Total Score	3.93 ± 2.88	4.00 ± 2.85	−0.07 ± 0.43	0.109	0.013
PHQ-9						
	Question 1	0.81 ± 0.72	0.78 ± 0.73	0.03 ± 0.17	0.083	0.015
	Question 2	0.85 ± 0.76	0.86 ± 0.75	−0.01 ± 0.17	0.566	0.002
	Question 3	0.78 ± 0.85	0.78 ± 0.86	0.00 ± 0.14	0.999	0.000
	Question 4	1.09 ± 0.78	1.08 ± 0.79	0.01 ± 0.10	0.320	0.005
	Question 5	0.75 ± 0.78	0.75 ± 0.79	0.00 ± 0.25	0.999	0.000
	Question 6	0.61 ± 0.76	0.64 ± 0.76	−0.03 ± 0.22	0.181	0.009
	Question 7	0.51 ± 0.75	0.53 ± 0.75	−0.02 ± 0.20	0.320	0.005
	Question 8	0.37 ± 0.68	0.37 ± 0.68	0.00 ± 0.00	1.000	0.000
	Question 9	0.10 ± 0.41	0.14 ± 0.45	−0.04 ± 0.20	0.065	0.020
	PHQ-9 Total Score	5.87 ± 4.57	5.93 ± 4.52	−0.06 ± 0.58	0.306	0.005
PHQ-15						
	Question 1	0.52 ± 0.64	0.60 ± 0.70	−0.08 ± 0.37	0.032*	0.023
	Question 2	0.74 ± 0.71	0.74 ± 0.71	0.00 ± 0.00	1.000	0.000
	Question 3	0.59 ± 0.64	0.63 ± 0.66	−0.04 ± 0.28	0.158	0.010
	Question 4	0.81 ± 0.65	0.81 ± 0.65	0.00 ± 0.00	1.000	0.000
	Question 5	0.16 ± 0.42	0.33 ± 0.59	−0.17 ± 0.47	0.001*	0.061
	Question 6	0.32 ± 0.55	0.49 ± 0.69	−0.17 ± 0.53	0.062	0.049
	Question 7	0.18 ± 0.31	0.30 ± 0.60	−0.12 ± 0.54	0.001*	0.057
	Question 8	0.30 ± 0.54	0.36 ± 0.58	−0.06 ± 0.28	0.063	0.023
	Question 9	0.21 ± 0.46	0.35 ± 0.58	−0.14 ± 0.43	0.001*	0.052
	Question 10	0.34 ± 0.59	0.48 ± 0.67	−0.14 ± 0.45	0.002*	0.047
	Question 11	0.46 ± 0.59	0.52 ± 0.61	−0.06 ± 0.28	0.063	0.023
	Question 12	0.89 ± 0.63	0.90 ± 0.64	−0.01 ± 0.10	0.320	0.015
	Question 13	0.49 ± 0.66	0.51 ± 0.67	−0.02 ± 0.20	0.320	0.030
	Question 14	0.83 ± 0.67	0.83 ± 0.67	0.00 ± 0.00	1.000	0.000
	Question 15	0.81 ± 0.62	0.81 ± 0.62	0.00 ± 0.00	1.000	0.000
	Total Score	5.91 ± 4.08	6.41 ± 4.12	−0.50 ± 1.01	0.097	0.121

PHQ-4: Patient Health Questionnaire-4; PHQ-9: Patient Health Questionnaire-9; PHQ-15: Patient Health Questionnaire-15; ES: Effect Size. Paired t-test; *p < 0.05.

The GAD-7 scale exhibited a significant difference for question 7, with a higher mean in the analog format (*p *< 0.05). However, the effect size was small (0.031) (Table 5).

**Table 5.** The assessment of the participants’ replies obtained from both the online and analog versions of the GAD-7 questionnaire with their effect sizes.

GAD-7	Online	Analog	Difference	*p*	ES
Question 1	0.89 ± 0.76	0.89 ± 0.76	0.00 ± 0.00	1.000	0.000
Question 2	0.74 ± 0.77	0.80 ± 0.82	−0.06 ± 0.24	0.014	0.031
Question 3	0.77 ± 0.74	0.80 ± 0.75	−0.03 ± 0.22	0.181	0.009
Question 4	0.82 ± 0.69	0.84 ± 0.71	−0.02 ± 0.14	0.158	0.010
Question 5	0.43 ± 0.64	0.48 ± 0.67	−0.05 ± 0.30	0.096	0.014
Question 6	0.66 ± 0.70	0.69 ± 0.73	−0.03 ± 0.17	0.083	0.015
Question 7	0.38 ± 0.58	0.44 ± 0.61	−0.06 ± 0.24	0.014*	0.031
Total Score	4.89 ± 3.94	4.94 ± 3.95	−0.05 ± 0.41	0.091	0.012

GAD-7: General Anxiety Disorder-7; ES: Effect Size. Paired t-test; *p < 0.05.

On the JFLS-8 and JFLS-20 scales, significant differences were noted for individual questions (*e.g.*, question 7 in JFLS-8 and questions 3, 4, 5 and 6 in JFLS-20). Analog responses consistently had small to medium effect sizes and higher means than online responses (Table 6).

**Table 6.** The assessment of the participants’ replies obtained from both the online and analog versions of the JFLS-8 and JFLS-20 questionnaires with their effect sizes.

Scale		Online	Analog	Difference	*p*	ES
JFLS-8						
	Question 1	1.16 ± 1.82	1.21 ± 1.80	−0.05 ± 0.22	0.025	0.026
	Question 2	0.50 ± 0.99	0.54 ± 1.02	−0.04 ± 0.20	0.045	0.020
	Question 3	0.25 ± 0.63	0.30 ± 0.64	−0.05 ± 0.22	0.025	0.026
	Question 4	0.41 ± 1.07	0.43 ± 1.08	−0.02 ± 0.14	0.158	0.010
	Question 5	0.39 ± 0.91	0.41 ± 0.91	−0.02 ± 0.14	0.158	0.010
	Question 6	0.85 ± 1.61	0.88 ± 1.60	−0.03 ± 0.17	0.083	0.015
	Question 7	0.49 ± 1.21	0.82 ± 1.47	−0.33 ± 0.97	0.001*	0.056
	Question 8	0.47 ± 0.98	0.54 ± 0.98	−0.07 ± 0.26	0.058	0.036
	Total Score	0.57 ± 0.62	0.64 ± 0.62	−0.07 ± 0.16	0.073	0.133
JFLS-20						
	Question 1	1.15 ± 2.08	1.16 ± 2.00	−0.01 ± 0.27	0.707	0.001
	Question 2	1.10 ± 1.98	1.14 ± 2.04	−0.04 ± 0.24	0.103	0.014
	Question 3	0.60 ± 1.33	0.69 ± 1.40	−0.09 ± 0.38	0.019*	0.028
	Question 4	0.61 ± 1.27	0.70 ± 1.28	−0.09 ± 0.38	0.019*	0.028
	Question 5	0.38 ± 0.99	0.42 ± 0.83	−0.13 ± 0.41	0.002*	0.072
	Question 6	0.31 ± 0.81	0.42 ± 0.83	−0.11 ± 0.31	0.010*	0.081
	Question 7	1.15 ± 2.20	1.16 ± 2.20	−0.01 ± 0.10	0.320	0.005
	Question 8	0.89 ± 1.72	0.90 ± 1.72	−0.01 ± 0.10	0.320	0.005
	Question 9	0.40 ± 0.94	0.50 ± 0.94	−0.10 ± 0.30	0.010	0.079
	Question 10	0.39 ± 0.94	0.48 ± 0.95	−0.09 ± 0.32	0.006	0.052
	Question 11	0.40 ± 0.89	0.45 ± 0.90	−0.05 ± 0.26	0.058	0.018
	Question 12	0.76 ± 1.60	0.76 ± 1.59	0.00 ± 0.14	1.000	0.000
	Question 13	0.46 ± 1.14	0.50 ± 1.15	−0.04 ± 0.24	0.103	0.014
	Question 14	0.55 ± 1.27	0.60 ± 1.27	−0.05 ± 0.26	0.058	0.018
	Question 15	0.45 ± 0.89	0.49 ± 0.90	−0.04 ± 0.24	0.103	0.014
	Question 16	0.37 ± 0.86	0.40 ± 0.87	−0.03 ± 0.17	0.083	0.015
	Question 17	0.32 ± 0.79	0.35 ± 0.80	−0.03 ± 0.17	0.083	0.015
	Question 18	0.51 ± 1.35	0.55 ± 1.34	−0.04 ± 0.20	0.055	0.020
	Question 19	0.55 ± 1.23	0.58 ± 1.23	−0.03 ± 0.17	0.083	0.015
	Question 20	1.12 ± 2.30	1.19 ± 2.28	−0.07 ± 0.26	0.058	0.036
	Global	0.58 ± 0.98	0.63 ± 0.96	−0.05 ± 0.11	0.062	0.092
	Mastication	0.69 ± 1.21	0.77 ± 1.21	−0.08 ± 0.18	0.074	0.108
	Mobility	0.71 ± 1.25	0.76 ± 1.23	−0.05 ± 0.12	0.101	0.091
	Communication	0.55 ± 0.94	0.59 ± 0.93	−0.04 ± 0.14	0.097	0.037
	Total Score	0.62 ± 1.02	0.69 ± 1.00	−0.07 ± 0.11	0.071	0.097

JFLS-8: Jaw Functional Limitation Scale-8; JFLS-20: Jaw Functional Limitation Scale-20; ES: Effect Size. Paired t-test; *p < 0.05.

While most comparisons between online and analog formats showed no significant differences, a few questions, such as GCPS question 7, GAD-7 question 7, and specific items in PHQ-4 and PHQ-15, exhibited differences that may arise from interpretation variability, content sensitivity, or format-related factors. Depending on how they are phrased, how comfortable the participant is, or the medium being used, subjective or delicate questions may generate somewhat different answers. Responses may also be influenced by variations in presentation, such as scrolling online versus looking at sequential paper layouts. Despite these differences having small effect sizes and limited practical impact, they highlight areas for improving scale design while affirming the overall reliability and validity of the online format.

For the OBC scale, questions 6, 9 and 12 exhibited significant differences, with the analog means exceeding the online means (*p* < 0.05). Effects varied between small to medium, keeping within acceptable ranges (Table 7).

**Table 7.** The assessment of the participants’ replies obtained from both the online and analog versions of the OBC questionnaire with their effect sizes.

OBC	Online	Analog	Difference	*p*	ES
Question 1	0.78 ± 1.26	0.84 ± 1.26	−0.06 ± 0.31	0.057	0.018
Question 2	0.85 ± 1.27	0.92 ± 1.22	−0.07 ± 0.52	0.179	0.009
Question 3	0.53 ± 0.89	0.60 ± 0.92	−0.07 ± 0.36	0.052	0.070
Question 4	0.77 ± 0.92	0.86 ± 0.95	−0.09 ± 0.40	0.058	0.024
Question 5	0.93 ± 1.06	0.97 ± 1.06	−0.04 ± 0.28	0.158	0.010
Question 6	0.67 ± 0.90	0.77 ± 0.93	−0.10 ± 0.44	0.025*	0.026
Question 7	0.51 ± 0.84	0.51 ± 0.84	0.00 ± 0.00	1.000	0.000
Question 8	0.44 ± 0.83	0.50 ± 0.86	−0.06 ± 0.31	0.057	0.042
Question 9	0.47 ± 0.83	0.59 ± 0.94	−0.12 ± 0.56	0.033*	0.046
Question 10	0.90 ± 1.07	0.95 ± 1.09	−0.05 ± 0.36	0.167	0.010
Question 11	0.52 ± 0.85	0.61 ± 0.92	−0.09 ± 0.47	0.060	0.044
Question 12	0.52 ± 0.81	0.61 ± 0.86	−0.09 ± 0.43	0.038*	0.022
Question 13	1.43 ± 1.10	1.43 ± 1.10	0.00 ± 0.00	1.000	0.000
Question 14	0.25 ± 0.63	0.31 ± 0.66	0.06 ± 0.83	0.063	0.092
Question 15	1.41 ± 1.18	1.41 ± 1.17	0.00 ± 0.14	1.000	0.000
Question 16	1.20 ± 1.19	1.20 ± 1.19	0.00 ± 0.00	1.000	0.000
Question 17	1.59 ± 1.09	1.58 ± 1.10	0.01 ± 0.17	0.566	0.002
Question 18	1.00 ± 1.03	1.03 ± 1.04	−0.03 ± 0.30	0.320	0.005
Question 19	1.23 ± 1.03	1.23 ± 1.03	0.00 ± 0.00	1.000	0.000
Question 20	1.61 ± 0.93	1.63 ± 0.95	−0.02 ± 0.14	0.158	0.010
Question 21	0.94 ± 1.05	1.02 ± 1.08	−0.08 ± 0.46	0.088	0.015
Total Score	18.55 ± 10.38	19.07 ± 10.32	−0.52 ± 1.05	0.094	0.051

OBC: Oral Behaviors Checklist; ES: Effect Size. Paired t-test; *p < 0.05.

These statistically significant, borderline small to medium sized effect sizes suggest that distinctions between the analog and the online versions are trivial and of no real practical importance. For instance, statistically significant differences in GCPS question 7 and GAD-7 question 7 had effect sizes below 0.060, suggesting minor variations in response styles rather than substantial disparities. Even medium-level effect sizes, such as in PHQ-15 questions, support the overall consistency of the constructs being measured without compromising scale reliability.

In fact, the absence of statistically significant variances in the vast majority of items and scales again provides strong support for the study’s primary objective: the validation of the equivalency between the traditional and the online versions of the DC/TMD Axis II Evaluation Questionnaires. The level of congruence even supports the robustness of scales, implying that they provide congruent and reliable results regardless of how they are administered.

These minor discrepancies between the two versions suggest that they are equally measuring the intended construct. The similarity likely reflects the strong design, coupled with pre-validation of the questionnaires. For clinical, as well as research use, where computers can greatly enhance accessibility, speed, and economy, the findings are of considerable import—demonstrating that diagnostic acuity need not be compromised in the administration of the digital versions.

While there were a few individual items that demonstrably had statistically significant differences, the actual effect sizes themselves remained trivial, suggesting minimal practical significance. These slight variations can be explained by the way that individuals respond to the digital in comparison to the paper medium, rather than significant differences in what the scales themselves are measuring. For that reason, the results confirm that the digital version can be considered a valid and reliable substitute of the original paper format, facilitating broader flexibility in administration without data loss.

These findings indicate the strong reliability and consistency of scales in the two formats of administration. High ICC values and strong Cronbach’s alpha scores confirm the instruments’ suitability for use in clinical and research settings and further show their reliability. Although some item-level differences were statistically significant, their effect sizes were small, suggesting limited practical impact, ensuring the overall reliability of the data across formats, confirming the feasibility of using the online format for accurate and consistent assessments in clinical and research settings.

## 4. Discussion

Sun *et al*. [31] demonstrated the reliability and validity of the PHQ-9 for assessing major depressive disorder (MDD) in a psychiatric hospital setting, reporting a Cronbach’s alpha of 0.892 and a moderate ICC of 0.594 when comparing PHQ-9 scores with Hamilton Depression Scale (HAMD) scores. The HAMD, a scale of depression symptoms that is rated by the clinician, was less concordant with the PHQ-9 probably because of variations in administration style and emphasis. Schiffman *et al*. [10] validated the DC/TMD diagnostic protocol, emphasizing its reliability and consistency across clinical and research settings [31]. In the present work, Cronbach’s alpha values all exceeded 0.700, while ICC values all remained above 0.950, supporting the strong reliability and equivalence of the DC/TMD-Axis II assessment in both the paper-and-pencil and the online formats. These results are in line with the findings of Sun *et al*. [31], further highlighting the robustness of well-validated assessment tools when administered in different formats [31]. While Sun *et al*. [31] observed moderate agreement between clinician-rated and self-reported measures, and Schiffman *et al*. [10] focused on diagnostic reliability in standardized settings, our study highlights the equivalency of online and analog formats, reinforcing the validity of digital adaptations for TMD assessment [10]. These comparisons, in total, lend support for wider generalizability and validity of standardized scales in different settings and formats of administration.

Andrade emphasized the built-in advantage of the use of online questionnaires—convenience, economy, and wider reach—just as much as the drawbacks, that is, response biases and challenges in representativeness of samples [14]. Geldsetzer *et al*. [39] emphasized the efficiency of online data collection, citing faster processing and reduced costs compared to analog methods, but acknowledged concerns about the accuracy of self-reported data. In our study, responses from the online version of the DC/TMD-Axis II Questionnaires were automatically recorded, while responses from the analog format required manual entry, reflecting the logistical benefits highlighted by Geldsetzer and Zagalaz-Anula [25, 39]. Moreover, in accordance with Andrade’s remark, the participants in our study opted to answer sensitive questions, including sexuality-related questions, via the online medium. Despite these logistical and contextual differences, our findings showed high reliability for both formats, with Cronbach’s alpha values exceeding 0.700 and ICC values consistently surpassing 0.950, validating the equivalency of online and analog methods for TMD assessment [14, 39].

Andrade outlined the advantages and the drawbacks of the utilization of the online questionnaire noting that despite clear advantages, they are also open to undefined sampling populations, self-selection bias, and greater missing data from unanswered questions [14]. In contrast, Geldsetzer *et al*. [39] conducted a large-scale online questionnaires during the COVID-19 pandemic, demonstrating the efficiency of online platforms in gathering rapid and diverse data from extensive populations, while highlighting limitations like potential response biases and representativeness [39]. In our study, the online format of the DC/TMD Axis II Questionnaires demonstrated comparable reliability to the analog version, with Cronbach’s alpha values consistently above 0.700 and ICC values exceeding 0.950, supporting strong agreement across formats. Unlike Andrade’s concern regarding reduced follow-up rates or missing data from electronic questionnaires, we found that the completion rates between the online questionnaires and the analog questionnaire were no different. This suggests that the validated nature of the questionnaires may help overcome these common challenges. Furthermore, consistent with Geldsetzer’s findings on the advantages of online data collection, the automated processes in our study facilitated rapid data recording and analysis, underscoring the practicality of the online format for TMDs assessments. These findings therefore confirm that, where they are valid, online surveys are a robust and stable alternative to the older paper-and-pencil method, being suitable for a wide range of clinical use and applications in research.

Mondal *et al*. [40] emphasize the benefits of using Google Forms in research, citing its ease of use, cost-free accessibility, and ability to quickly reach a large number of respondents, all of which were observed in our study during data collection and analysis. It has practical advice on converting paper questionnaires to electronic ones, illustrated by examples of use in medical studies [40]. Similar results were observed in this study during data collection and analysis. Similarly, our study verified that the DC/TMD Axis II Evaluation Questionnaire in its online format possessed equal reliability and consistency when compared to the standard analog technique. Mondal *et al*. [40] also highlighted that analog questionnaires are more inclusive of populations with limited internet access or technological proficiency, a consideration that aligns with Andrade *et al*.’s [14] observation that analog methods allow face-to-face interactions, enabling researchers to clarify questions and improve data quality [40]. While the findings indicated no meaningful disparity between the analog questionnaire and the online questionnaire formats, they do indicate some of the practical advantages of the analog questionnaires—on the part of clearly identifiable demographic groups. These results, in general, reflect the flexibility of the two methods and emphasize the necessity of selecting the optimal format based on the circumstances of the research as well as the nature of the potential population.

Analog questionnaires can offer special advantages by doing away with common technological pitfalls of device compatibility, browser issues, or unreliable connections, therefore simplifying the process of gathering data. For other participants, however, especially those less familiar with computers, completing a questionnaire by paper may also be less threatening and safer—potentially evoking candid responses to sensitive or private questions because of fewer data safety or privacy issues. Non-analog formats are, however, not without potential disadvantages. For example, the investigator’s presence may inadvertently influence participants towards offering socially acceptable responses. By comparison, the online questionnaires may be at risk of self-selection bias, in that they will tend to attract the young or highly experienced in technologies—potentially rendering the sample less representative [41, 42, 43, 44, 45, 46, 47]. While the analog questionnaires allow researchers to provide real-time explanation and verification of thoroughness, the online questionnaires are at greater risk of incompletely filled-in or hasty responses, especially if the participants are distracted or multi-tasking. An awareness of such variations in methods and potential sources of biases are crucial in aiding appropriate interpretation of the findings to provide that the results can be generalized beyond the sample.

When evaluating internal consistency, a Cronbach’s alpha value of 0.700 or above is generally considered the minimum acceptable threshold [37]. Each of the scales of the present work met or exceeded this criterion, attesting to acceptable reliability for the paper and the online formats. Robust performances by Cronbach’s alpha, as well as the intraclass correlation coefficient (ICC), further attest to the reliability, as well as the consistency, of the DC/TMD Axis II Questionnaires, regardless of how they were administered. The paired *t*-test results further supported the equivalence of the two formats, as no statistically significant differences were found between them. While Bland-Altman analysis can commonly be applied for the determination of agreement between two continuous methods of measurement, we did not use this in the present work. Instead, we focused upon general reliability in the form of ICC, in combination with Cronbach’s alpha—both well-established within the validation of questionnaires. The paired *t*-test results, in combination with calculation of the effect size, further attested that the two formats had equivalency, in accordance with the primary focus of the present work. Since our primary focus of the present work was validation of general reliability, with equivalence between formats, in place of the determination of the item-by-item agreement, the Bland-Altman analysis of this work was considered to be out of scope. It can, however, be the subject of future work, in terms of determining the individual-level agreement, in greater specificity, complementing the broader reliability markers that we applied here.

In validation studies within the literature, it has been observed that questionnaires have been conducted at intervals ranging from 1 day to 5 years [23, 24, 25, 46]. According to COSMIN guidelines for reliability studies, we chose a one-week interval in the present study in an attempt to find a balance between reducing immediate recall bias and limiting the chances of participants’ health status change that might influence their responses. The choice of time interval for test-retest evaluation corresponds to common procedures in questioning validation and offers, at the same time, a fair time frame for the determination of response consistency. It is, however, conceivable that the participants will remember their former responses—particularly in items that are of personal significance or highly memorable—thus compromising the independence of the response. Future studies can, in that sense, try different intervals of time, shorter or longer, in order to find a better balance between the risk of the recall bias and the requirement to assure reliability.

From the clinical point of view, the findings of the present work support the fact that the analog and the online versions of the questionnaire are of equal reliability. It therefore follows that online versions can be introduced in clinical work with assurance in aiding work-flow, in reducing administration, and in enhancing the accessibility of assessment, without loss of the quality or accuracy of the data thus obtained.

Note that the reliability of all the reliability studies largely depends on the sample used. So that the outcomes be generalizable in the broader population, participants must be representatively and randomly selected. For the present work, a randomized sampling plan has been made analogous to other works [25, 48, 49], in an effort to maximize validity in addition to the generalizability of the findings.

The DC/TMD Axis II Questionnaires are well-established instruments that enjoy a satisfactory record of reliability and validity, for which several validation studies provide support. Their broad utility has further corroboration in the fact that they are authored in multiple languages, hence increasing their use in different populations [10]. With the growing emergence of digital instruments, the employment of the administration of the tests in the online format acquires increasing importance within the clinical environment as well as in the field of research [41]. The work at hand offers crucial evidence, by demonstrating that the DC/TMD Axis II in the online format are efficient, trustworthy alternatives of the administration in paper-and-pencil. 100 participants formed the population, based on COSMIN recommendations, which specify the same number as the minimal for validation studies. Although satisfactory in the quantification of reliability as well as internal consistency, the relatively limited, homogeneous, largely young, educated population does not provide generalizable outcomes, mainly in populations of different degrees of technological knowledge.

The ability to pose a sizable number of questions to a large population is what makes this study novel. Our study thoroughly evaluated both online and analog formats of all diagnostic questionnaires at the same time, whereas previous research has only used a small number of DC/TMD Axis II evaluation tools to diagnose TMDs [30, 31, 33]. This study is unique in that it is the first to validate the online versions of a comprehensive set of questionnaires intended to evaluate TMDs. As a result, this study will significantly advance the body of current literature. Future studies should consider larger, multi-center samples to include a variety of demographics and examine the effects of factors such as age, educational attainment, and digital literacy on responses. Furthermore, more detailed results might be obtained by including both TMD patients and healthy controls.

This study had some limitations. While the use of DC/TMD criteria in an online format may show consistent diagnostic outcomes when compared to diagnoses made in clinical settings. Nevertheless, our investigation was conducted using a sample of randomly selected individuals who did not undergo diagnostic procedures. Consistency between the analog and the online versions of the questionnaires, by general acceptance, was compared. Future research could involve diagnostic investigations including individuals with TMDs and a control group of healthy individuals. Ensuring sufficient sample sizes is important for the conduct of validation studies. COSMIN recommendations suggest that at least 100 participants and 5 times the number of questionnaire items for factor analysis be considered appropriate [36]. This study was conducted with 100 patients but, it is significant for future investigations to consider larger sample sizes. Additionally, future studies should explore further validity measures, such as construct and criterion validity, to more robustly establish the equivalency of the online format in broader clinical contexts, as this was beyond the primary scope of the current research.

## 5. Conclusions

In conclusion, the online form of the DC/TMD Axis II Assessment Questionnaires proved itself to be a consistent and reliable tool for assessing the temporomandibular disorder’s symptoms. High Intraclass Correlation Coefficients (ICC) (all above 0.950) and Cronbach’s alpha coefficients (above 0.700) verify the high agreement and internal consistency of the online version. This version of questionnaires can be used to define the TMDs.

The online modality offers a variety of advantages in the clinical setting, including ease in the administration, rapid data collection, and increased accessibility. For these reasons, it can prove a very useful tool in initial screenings, follow-up, and incorporation in telemedicine services, where quick and efficient diagnostic instruments are particularly crucial. The online version’s usefulness is supported by the equivalency between its analog and online formats, which encourages its wider use in a variety of clinical and research settings.

Future research should also assess the reliability of the online format over different time periods and compare its diagnostic accuracy to clinical diagnoses. These guidelines would increase the online version’s applicability in different healthcare settings and validate it even more.
